# Significance of detecting cardiac troponin I and creatine kinase MB in critically Ill children without primary cardiac illness

**DOI:** 10.3389/fped.2024.1445651

**Published:** 2024-09-02

**Authors:** Yangyang Zhang, Yinyin Cao, Yi Xin, Yongming Liu

**Affiliations:** ^1^ Department of Pediatrics, Qingdao University Medical College Affiliated Yantai Yuhuangding Hospital, Yantai, Shandong, China; ^2^ Clinical Laboratory, Qingdao University Medical College Affiliated Yantai Yuhuangding Hospital, Yantai, Shandong, China

**Keywords:** primary cardiac disease, critical illness, myocardial injury, cardiac troponin I, creatine kinase, children

## Abstract

**Objective:**

To investigate the incidence of myocardial injury in children with critically ill children without primary cardiac disease and the association between elevated cardiac troponin I (cTnl) and creatine kinase MB (CK-MB) concentrations and disease progression and prognosis to guide early treatment.

**Methods:**

The serum cTnI and CK-MB concentrations of 292 children with critically ill children without primary cardiac disease in Yantai Yuhuangding Hospital between January 2021 and January 2024 were retrospectively analyzed within 24 h after entering the Pediatric Intensive Care Unit (PICU). The children were divided into normal and abnormal groups according to the myocardial marker results. The abnormal group was further divided into the cTnI-elevated, CK-MB-elevated, single-elevated (cTnI- or CK-MB-elevated) and double-elevated (cTnI- and CK-MB-elevated) groups. The differences in the clinical indicators and their relationships with prognosis for the groups were compared.

**Results:**

The incidence of myocardial injury among the critically ill children without primary cardiac disease was 55.1%. The incidence of myocardial injury in children with infectious diarrhea combined with moderate and severe dehydration reached 85.19%. The pediatric critical illness score; frequency of use of vasoactive drugs; hypotension, shock, heart failure, respiratory failure, and multiple organ dysfunction syndrome; and mortality indexes differed significantly for the normal and abnormal myocardial marker groups (*P* < 0.05). The single-elevated and normal groups only showed a difference in mortality (*P* < 0.017). The cTnI and CK-MB concentrations were negatively correlated with prognosis (*P* < 0.01).

**Conclusion:**

Myocardial injury, as evidenced by elevated cardiac biomarkers, is common in critically ill children without primary cardiac illness. cTnI and CK-MB are associated with outcomes. Shock, heart failure, and multiple organ dysfunction syndromes are independently associated with simultaneous elevations of CK-MB and cTnI concentrations. Further prospective studies are needed to elucidate the clinical utility of these biomarkers.

## 1 Introduction

Myocardial injury can be caused by a variety of critical illness. In recent years, myocardial injury has been found in critically ill patients without primary heart disease, including those with severe pneumonia; severe hand, foot, and mouth disease; sepsis; septic shock; novel coronavirus infection; adenoviral infection; Epstein-Barr virus infection; coxsackie virus infection; herpes virus infection; influenza; intracranial hemorrhage; and multiple organ failure (1–3). Studies have shown that the incidence of myocardial injury is as high as 88% among children with septic shock (4) and 50% among patients with severe coronavirus disease 2019 (5). Clinically, the symptoms of myocardial injury in children are not typical and are often masked by the symptoms of the primary disease, followed by symptoms of cardiac insufficiency such as low heart sound, hypotension, and early shock, among others. This can lead to severe heart failure, multiple organ failure, and death. Therefore, clinicians need to detect myocardial injury early to improve prognosis, particularly in critically ill children without primary cardiac disease. Studies have shown that myocardial damage in children with sepsis can cause abnormal cardiac systolic function, and clinicians can evaluate the prognosis of children with myocardial damage markers (6).

Various components of myocardial cells are released into the blood after myocardial cell injury. Studies have found that creatine kinase isoenzyme (CK-MB) and cardiac troponin (cTnI) concentrations are more specific and sensitive in detecting myocardial injury among them (7–10). CK-MB and cTnI are associated with prognosis (11–14). The changes in the concentrations of cTnI and CK-MB have an early onset, long duration, high sensitivity, and strong specificity after myocardial injury and are currently used for routine testing for suspected myocardial injury in clinical practice. Based on the combined detection of cTnI and CK-MB concentrations in 292 critically ill children without primary cardiac disease, this study aimed to determine the incidence of myocardial injury in critically ill children without primary cardiac disease; explore the associations between myocardial injury and disease progression and prognosis; and emphasize the need for early detection of myocardial injury, timely treatment, and improvement of prognosis. The insights will guide the treatment of children with critical illness.

## 2 Materials and methods

### 2.1 General information

A total of 292 patients were admitted to the Pediatric Intensive Care Unit (PICU) of Yantai Yuhuangding Hospital between January 2021 and January 2024; these excluded those with primary cardiac disease and musculoskeletal system disease when admitted to the PICU, such as viral myocarditis, acute and chronic heart disease, coronary disease, arrhythmia, chest trauma, cardiopulmonary resuscitation, myodystrophy, and post-defibrillation. Of the included patients, 176 were males, and 116 were females. Their ages ranged from 29 days to 6 years, with a mean of 37.38 months. The primary cases included 127 cases of respiratory diseases (including severe pneumonia, severe bronchitis, novel coronavirus infection, infectious laryngitis, asthma, and drowning); 53 cases of intracranial lesions (including intracranial infection, intracranial hemorrhage, and epilepsy); 30 cases of poisoning (including from drugs and poisons); 27 cases of infectious diarrhea combined with moderate and severe dehydration; 17 cases of severe hand, foot and mouth disease; and 38 other cases (including blood system disease, tumor, genetic metabolic disease, heat syndrome, skin and soft tissue infection, and unclear diagnoses).

### 2.2 Ethics

This study meets the standards of medical ethics and was approved by the Ethics Committee of Yantai Yuhuangding Hospital (Approval number: 2024-394).

### 2.3 Method

The serum cTnI and CK-MB concentrations of the 292 critically ill children without primary cardiac disease were measured using chemiluminescence within 24 h after admission to the PICU. The assays were the same for all patients included. The normal concentrations of cTnI and CK-MB are 0–0.06 ng/ml and 0–5 ng/ml, respectively. The pediatric critical illness score (PCIS) is based on the following 10 physiological indicators introduced by the First Aid Group, Pediatrics Branch, and Chinese Medical Association: heart rate, systolic blood pressure, respiratory rate, oxygen partial pressure, pH, serum sodium, serum potassium, creatinine or urea nitrogen, hemoglobin, and gastrointestinal system. Lower scores are associated with a more critical condition. It was calculated according to the clinical data within 24 h after admission to the PICU. The hemodynamic changes, cardiac insufficiency, and multiple organ dysfunction syndromes (MODSs) were also assessed and recorded during the disease course. The MODSs were diagnosed according to the diagnostic criteria formulated by the First Aid Group, Pediatric Society, and Chinese Medical Association. Shock was diagnosed according to the consensus on circulatory shock and hemodynamic monitoring by the Task Force of the European Society of Intensive Care Medicine. Hypotension was diagnosed according to the age-related criteria provided by the Pediatric Advanced Life Support Course. Heart failure was diagnosed according to the children's heart failure diagnosis and treatment recommendations (2020 revision) formulated by the Cardiology Group, Pediatric Society, and Chinese Medical Association. Respiratory failure was diagnosed according to the Guidelines for Pediatric Respiratory Failure formulated by the Extracorporeal Life Support Organization (ELSO). The primary outcome was defined as the incidence of myocardial injury based on elevated myocardial biomarkers. The secondary outcomes were defined as associations between elevated cardiac biomarkers and clinical outcomes such as mortality, MODS, heart failure, respiratory failure, PCIS, shock, and vasoactive use.

### 2.4 Groups

The patients were grouped into the normal and abnormal myocardial marker groups based on their cTnI and CK-MB concentrations. The abnormal myocardial marker group was further divided into the cTnI-elevated, CK-MB-elevated, single-elevated (cTnI or CK-MB elevated), and double-elevated (cTnI and CK-MB elevated) groups. According to the prognosis, the patients were divided into two groups: improvement (including cure) (grade 2) group and treatment failure (grade 1) group. The patients in the improvement group survived, showed improvement, and were transferred from the PICU to the general ward. The patients in the treatment failure group included patients who gave up on treatment for various reasons and patients who died.

### 2.5 Statistical analysis

SPSS software was used for all statistical analyses. The measurement data were analyzed using the *t*-test, analysis of variance (ANOVA), and non-parametric statistical methods to determine the differences between the two groups (*P* < 0.05 was considered statistically significant). The Chi-squared test was used to compare the counting data (*P* < 0.05 was considered statistically significant). The Chi-squared test segmentation was used to determine the intra-group differences (modified parameter *P* < 0.017 was considered statistically significant). The association between the biomarkers and outcomes was assessed using regression analysis (*P* < 0.05 denoted statistical significance).

## 3 Results

### 3.1 Results of myocardial markers detection

**Figure 1:**
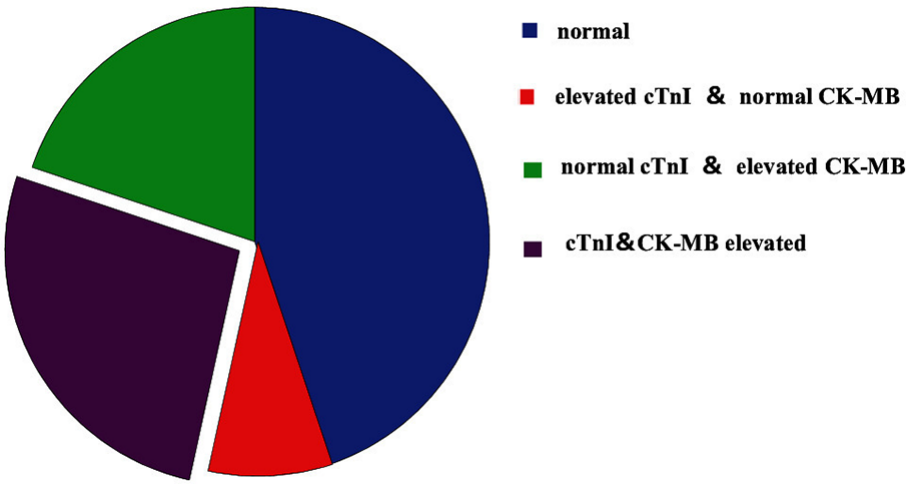
Results of myocardial markers.

Of the 292 cases, 161 had abnormal concentrations of cTnI and CK-MB (55.14%), with mean values of 2.56 ± 12.35 ng/ml and 55.01 ± 197.13 ng/ml; 103 cases (35.27%) had elevated cTnI concentrations (mean: 4.00 ± 15.28 ng/ml), 136 cases (46.57%) had elevated CK-MB concentrations (mean: 64.59 ± 213.21 ng/ml), 83 cases had elevated concentrations of cTnI (mean: 0.16 ± 0.65 ng/ml) or CK-MB (mean: 13.19 ± 19.89 ng/ml), 78 cases had elevated concentrations of both cTnI (5.12 ± 17.42 ng/ml) and CK-MB (99.51 ± 276.46 ng/ml) (Figure 1).

### 3.2 Incidence of myocardial injury among patients with different diseases

**Figure 2:**
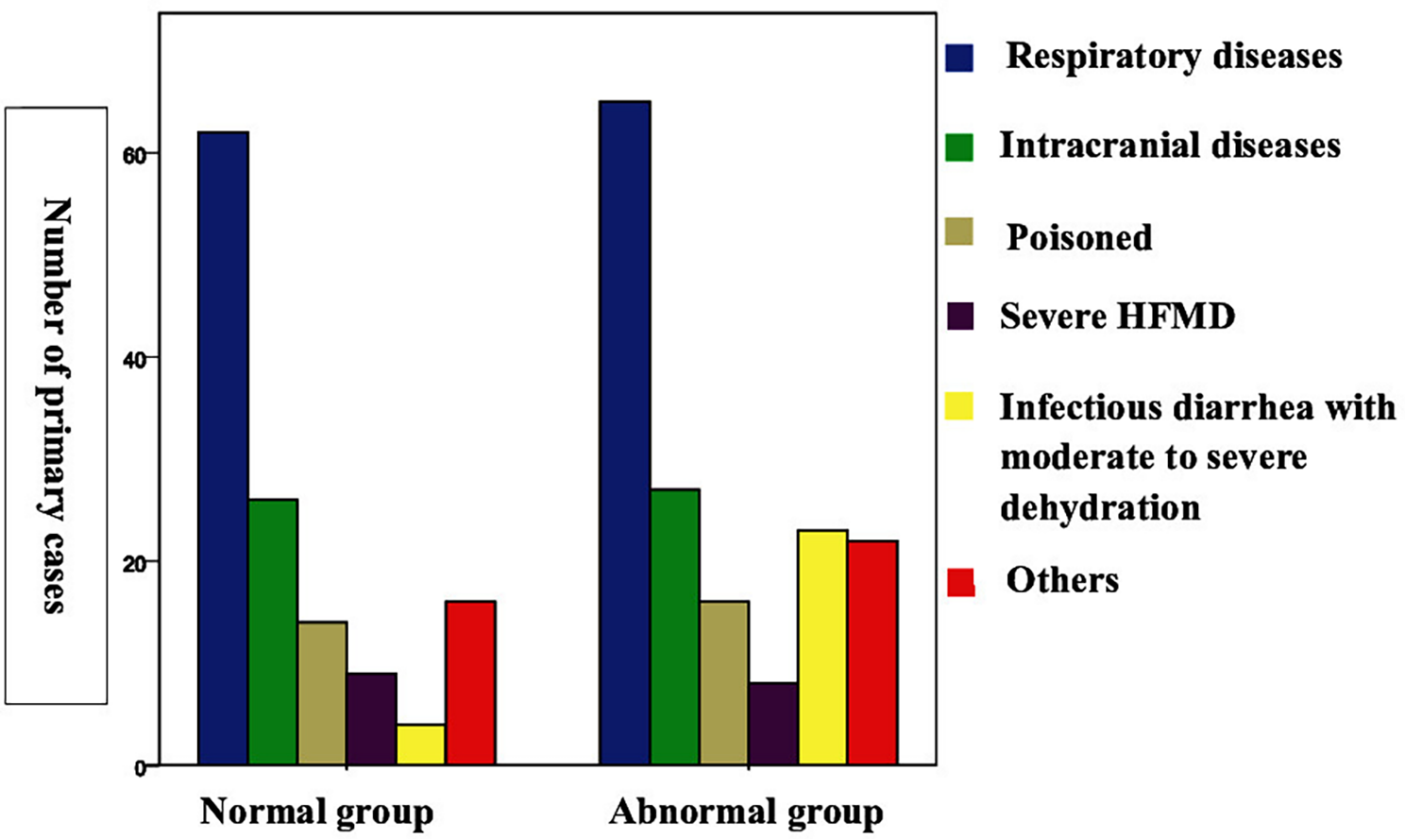
Myocardial injury in different diseases.

The incidence of myocardial injury among the patients with different diseases were as follows: infectious diarrhea with moderate to severe dehydration: 23/27 cases (85.19%); respiratory diseases: 65/127 cases (51.18%); intracranial lesions: 27/53 cases (50.94%); poisoning: 16/30 cases (53.33%); severe hand, foot, and mouth disease: 8/17 cases (47.06%); and others: 22/38 cases (57.89%) (Figure 2; Table 1).

**Table 1:** Primary disease composition of normal and abnormal myocardial markers.

		Primary disease						Total
		Respiratory diseases	Nervous system disease	Poisoning	Hand-foot-and-mouth disease	Diarrhea with moderate to severe dehydration	Others	
Normal	*n*	62	26	14	9	4	16	131
Group	%	48.80	49.10	46.70	52.90	14.80	42.10	44.90
Abnormal	*n*	65	27	16	8	23	22	161
Group	%	51.20	50.90	53.30	47.10	85.20	57.90	55.10
Total	*n*	127	53	30	17	27	38	292

### 3.3 Comparison of myocardial markers of the groups

However, there were statistically significant differences in the PCIS (80.08 ± 8.92 vs. 75.90 ± 8.54); prevalence of hypotension (15.3% vs. 31.1%), shock (13.0% vs. 30.4%), use of vasoactive drugs (including epinephrine, norepinephrine, dopamine, dobutamine, and milrinone) (19.8% vs. 39.8%), heart failure (8.4% vs. 21.7%), respiratory failure (58.8% vs. 73.9%), MODS (41.2% vs. 67.1%), and mortality rate (7.6% vs. 22.4%) between the normal and abnormal myocardial biomarker groups (Table 2).

**Table 2:** Comparison of the clinical indicators of the normal and abnormal myocardial marker group.

Clinical indicators	Normal group	Abnormal group	*χ*^2^/*t*	*P*
	(*n* = 131)	(*n* = 161)		
Age (m)	40.665 ± 41.506	34.713 ± 43.443	1.188	0.236
Gender: male, *n* (%)	87 (66.412)	89 (55.280)	3.288	0.070
Female, *n* (%)	44 (33.588)	72 (44.720)		
Hypotension, *n* (%)	20 (15.267)	50 (31.056)*	9.879	0.002
Shock, *n* (%)	17 (12.977)	49 (30.435)*	12.58	<0.001
Vasoactive drugs, *n* (%)	26 (19.847)	64 (39.752)*	13.42	<0.001
Heart failure, *n* (%)	11 (8.397)	35 (21.739)*	9.688	0.002
Respiratory failure, *n* (%)	77 (58.779)	119 (73.913)*	7.497	0.006
MODS, *n* (%)	54 (41.221)	108 (67.081)*	19.55	<0.001
Death, *n* (%)	10 (7.634)	36 (22.360)*	33.79	<0.001
PCIS	80.081 ± 8.915	75.899 ± 8.540	4.074	<0.001

* Compared with the normal group, *P* < 0.01

There were no significant differences in age, gender, PCIS, hypotension, shock, use of vasoactive drugs, heart failure, respiratory failure, MODS, and mortality rate between the cTnI- and CK-MB-elevated groups (*P* > 0.05) (Table 3).

**Table 3:** Comparison of the clinical indicators of the elevated cTnI and CK-MB groups.

Clinical indicators	Elevated cTnI group	Elevated CK-MB group	*χ*^2^/*t*	*P*
	(*n* = 103)	(*n* = 136)		
Age (m)	36.861 ± 45.478	32.352 ± 40.540	−0.809	0.420
Gender: male, *n* (%)	57 (55.340)	76 (55.882)	0.007	0.933
Female, *n* (%)	46 (44.660)	60 (44.118)		
Hypotension, *n* (%)	41 (39.806)	41 (30.147)	2.426	0.119
Shock, *n* (%)	40 (38.835)	45 (33.088)	0.845	0.358
Vasoactive drugs, *n* (%)	48 (46.602)	55 (40.441)	0.907	0.341
Heart failure, *n* (%)	29 (28.155)	32 (23.529)	0.660	0.417
Respiratory failure, *n* (%)	78 (75.728)	101 (74.265)	0.067	0.796
MODS, *n* (%)	79 (76.699)	94 (69.118)	1.685	0.194
Death, *n* (%)	28 (27.184)	33 (24.265)	0.439	0.803
PCIS	74.928 ± 8.011	75.252 ± 8.679	0.292	0.772

Compared with the single-elevated group, the double-elevated group had a higher risk of hypotension (41.0% vs. 21.7%; chi-squared = 7.02; *P* = 0.008), shock (46.2% vs. 15.7%; chi-squared = 17.66; *P* < 0.001), the use of vasoactive drugs (50.0% vs. 30.1%; chi-squared = 6.64; *P* = 0.001), heart failure (33.3% vs. 10.8%; chi-squared = 11.96; *P* = 0.001) and MODS (83.3% vs. 51.8%; chi-squared = 18.10; *P* < 0.001). The mortality rate (32.1% vs. 13.3%; chi-squared = 10.32; *P* = 0.006) was also significantly higher, and the PCIS (73.49 ± 7.85 vs. 78.18 ± 8.58; *t* = 4.68; *P* = 0.001) was lower. The group had a significantly higher prevalence of hypotension (41.0% vs. 15.3%; chi-squared = 17.36; *P* < 0.001), shock (46.2% vs. 13.0%; chi-squared = 28.43, *P* < 0.001), use of vasoactive drugs (50.0% vs. 19.8%; chi-squared = 20.74; *P* < 0.001), heart failure (33.3% vs. 8.4%; chi-squared = 20.87; *P* < 0.001), respiratory failure (76.9% vs. 58.8%; chi-squared = 7.13; *P* = 0.008), and MODS (83.3% vs. 41.2%; chi-squared = 35.36; *P* < 0.001), and a higher mortality rate (32.1% vs. 7.6%; chi-squared = 42.59; *P* < 0.001) than the normal group. The double-elevated group had a significantly lower PCIS than the normal group (73.49 ± 7.85 vs. 80.08 ± 8.92; *t* = 6.59; *P* < 0.001). There was also a significant difference between the mortality rates of the single-elevated and normal groups (7.6% vs. 13.3%; chi-squared = 13.57; *P* = 0.001), but no significant differences between the other indicators. There were no significant differences in age and gender between the normal group, single-elevated, and double-elevated groups (*P* > 0.05) (Table 4).

**Table 4:** Comparison of the clinical indicators of the normal, single-elevated, and double-elevated groups.

Clinical indicators	Normal group	Single elevated group	Double elevated group	*χ*^2^/*F*	*P*
	(*n* = 131)	(*n* = 83)	(*n* = 78)		
Age (m)	40.665 ± 41.506	35.912 ± 45.478	33.441 ± 41.424	0.771	0.464
Gender: male, *n* (%)	87 (66.412)	45 (54.217)	44 (56.410)	3.819	0.148
Female, *n* (%)	44 (33.588)	38 (45.783)	34 (43.590)		
Hypotension, *n* (%)	20 (15.267)	18 (21.687)	32 (41.026)*^,^^#^	18.13	<0.001
Shock, *n* (%)	17 (12.977)	13 (15.663)	36 (46.154)*^,^^#^	33.95	<0.001
Vasoactive drugs, *n* (%)	26 (19.847)	25 (30.120)	39 (50.000)*^,^^#^	20.87	<0.001
Heart failure, *n* (%)	11 (8.397)	9 (10.843)	26 (33.333)*^,^^#^	25.01	<0.001
Respiratory failure, *n* (%)	77 (58.779)	59 (71.084)	60 (76.923)*	8.118	0.017
MODS, *n* (%)	54 (41.221)	43 (51.807)	65 (83.333)*^,^^#^	35.73	<0.001
Death, *n* (%)	10 (7.634)	11 (13.253)	25 (32.051)*^,^^#^	45.95	<0.001
Pcis	80.081 ± 8.915	78.167 ± 8.582	73.493 ± 7.849*^,^^#^	14.64	<0.001

* Compared with the normal group, the difference was statistically significant *P* < 0.017 (Chi-squared test segmentation correction).

^#^ Compared with the single elevated group, the difference was statistically significant *P* < 0.017.

### 3.4 Relationships between cTnI, CK-MB, and prognosis

**Figure 3:**
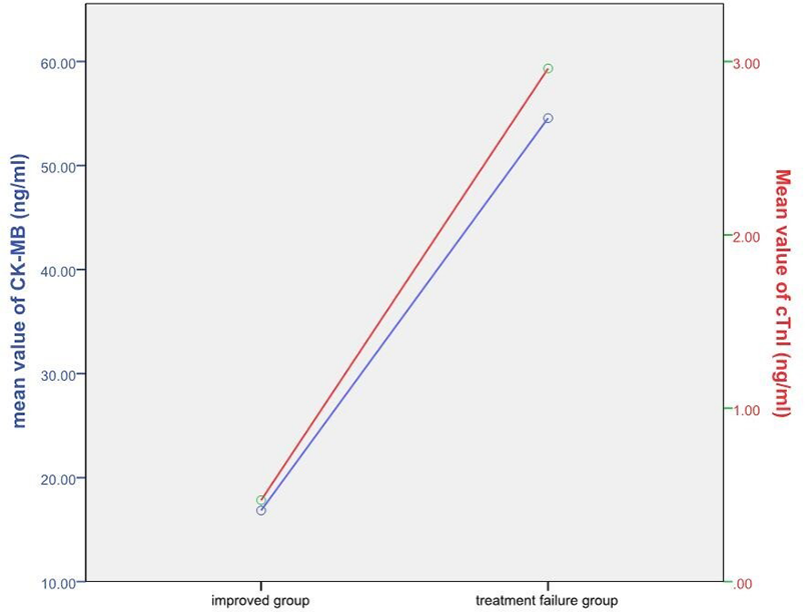
Relationship between cTnI, CK-MB and prognosis. The red line represents cTnI and the blue line represents CK-MB.

There were significant differences between the cTnI (0.24 ± 1.06 vs. 2.96 ± 14.53) and CK-MB (13.27 ± 64.93 vs. 54.56 ± 220.29) concentrations of the improved and the treatment failure group (*P* < 0.001) (Figure 3). Table 5 shows the significant negative correlations of the cTnI and CK-MB concentrations with prognosis. Univariate logistic regression analysis showed that elevation of CK-MB concentration, elevation of cTnI concentration, elevation of CK-MB or cTnI concentration, and simultaneous elevations of CK-MB and cTnI concentrations were independent risk factors for prognosis (*P* < 0.05) (Table 6). Multivariate regression analysis showed that shock, heart failure, and MODS were independently associated with mortality while the simultaneous elevations of CK-MB and cTnI concentrations was not (*P* < 0.05). But it was independently associated with shock, heart failure, and MODS (*P* < 0.05). By multivariate regression analysis after removal the independent variables of shock, heart failure and MODS, we found that the simultaneous elevations of CK-MB and cTnI concentrations is an independent risk factor for mortality (*P* < 0.05).

**Table 5:** Correlations of CK-MB and cTnI concentrations with the prognosis of myocardial injury.

Indicators	Spearman's Rho correlation coefficient	*P*
CK-MB and prognosis	−0.218	<0.001
cTnI and prognosis	−0.198	<0.001

**Table 6:** Logistic regression analysis.

Dependent variable: prognosis						
Independent variable	B	S.E,	Wals	df	Sig.	Exp (B)
cTnI elevated	0.746	0.252	8.799	1	0.003	2.109
Constant	−0.765	0.156	24.00	1	<0.001	0.465
CKMB elevated	0.905	0.247	13.41	1	<0.001	2.472
Constant	−0.934	0.178	27.58	1	<0.001	0.393
CK-MB/cTnI elevated			15.22	2	<0.001	
Single elevated	0.644	0.298	4.663	1	0.031	1.903
Double elevated	1.163	0.301	14.94	1	<0.001	3.200
Constant	−1.009	0.197	26.11	1	<0.001	0.365

## 4 Analysis and discussion

### 4.1 Incidence and possible mechanism of myocardial injury

In this study, 161 of 292 children had abnormal myocardial markers, suggesting that the incidence of myocardial injury in critically ill children without primary cardiac disease was as high as 55.14%. This is consistent with the reports of related research (1–5). The possible mechanisms of myocardial injury in critically ill children without primary cardiac disease may include the direct injury of toxins, inflammatory injury, mitochondrial dysfunction, myocardial apoptosis, hypoxia and ischemia caused by disease, and imbalance of oxygen supply and demand under stress and high metabolic conditions (15–19). The exact mechanism requires further study.

### 4.2 Disease spectrum of myocardial injury

In this study, myocardial injury in critically ill children without primary cardiac disease was often secondary to respiratory diseases; intracranial lesions; poisoning; infectious diarrhea combined with moderate and severe dehydration; and severe hand, foot, and mouth disease. The incidence of myocardial injury in children with infectious diarrhea combined with moderate and severe dehydration reached 85.19%. All patients with infectious diarrhea admitted to PICU were considered to have severe infections with severe complications such as dehydration, hypovolemia, sepsis, and septic shock. Therefore, they were more susceptible to myocardial injury. However, research on this is limited, and further research is needed to confirm it (20).

### 4.3 Comparison of normal and abnormal myocardial marker groups

Studies have shown that cTnI can be used as a marker of severe hypoxia and ischemic myocardial damage in newborn dogs (21). cTnI and CK-MB concentrations have considerable value for predicting adverse outcomes among patients with HCM (22). In this study, cardiac dysfunction (hypotension, shock, use of vasoactive drugs), heart failure, respiratory failure, and MODS were more likely to occur in patients in the abnormal myocardial marker group (all included patients except those with normal CK-MB and cTnI concentrations). These are associated with a higher mortality rate. Early detection of myocardial injury using myocardial markers facilitates the assessment of cardiac function, severity of illness, and prognosis.

### 4.4 Comparison of the cTnI- and CK-MB-elevated groups

In this study, the elevations of cTnI and CK-MB concentrations were equally important in assessing cardiac function, incidence of respiratory failure, MODS, severity of illness, and mortality in children with myocardial injury. Both are suitable for the early diagnosis and follow-up of myocardial injury in critically ill children without primary cardiac disease. Combined with the differences between their sensitivities and specificities for the detection of myocardial injury, CK-MB and cTnI should be jointly detected in clinical practice to improve the early detection rate of myocardial injury and prognosis, and prevent the occurrence of shock, heart failure, and MODS.

### 4.5 Comparison of myocardial markers of the normal, single-elevated, and double-elevated groups

The three groups had significant differences in the clinical indicators. The chi-squared test showed that the difference was more significant than that between the abnormal and normal groups. It indicated that the double-elevated group (the simultaneous elevations of the concentrations of cTnI and CK-MB) plays an important role. Pairwise comparison of the groups showed that the mortality rate of the single-elevated group (an elevated concentration of cTnI or CK-MB)was significantly higher than that of the normal group. This suggests that CK-MB and cTnI concentrations may be related to prognosis, and we confirmed this below. Meanwhile, the risks of hemodynamic changes, cardiac insufficiency, and MODS did not increase with an increase in the concentration of a single marker when CK-MB and cTnI were both assessed. In clinical practice, the elevation of the concentration of CK-MB or cTnI when both are assessed may not be associated with the incidence of hemodynamic instability, heart failure, and MODS, among others. However, it may be related to a poorer prognosis than that of patients with normal myocardial markers. If the concentrations of both markers were elevated, the risks of hemodynamic changes, cardiac insufficiency, and MODS increased significantly, and the mortality rate was 4.22 times that of children with normal myocardial markers and 2.41 times that of children with the elevation of the concentration of a single myocardial marker. Therefore, the elevations of the concentrations of CK-MB and cTnI should be combined to evaluate the progression and prognosis of diseases.

### 4.6 Relationships between cTnI, CK-MB, and prognosis

In this study, the CK-MB and cTnI concentrations of children who died were significantly higher than those of the children in the improvement group, and the differences were statistically significant. Correlation analysis showed that the CK-MB (rs = −0.218) and cTnI (rs = −0.198) concentrations were significantly negatively correlated with prognosis; higher CK-MB and cTnI concentrations were associated with a worse prognosis. This is consistent with the reports of several studies (12, 23, 24). Univariate logistic regression showed that the elevations of cTnI and CK-MB concentrations are independent risk factors for poor prognosis but this was not demonstrated by multivariate regression analysis. Relative to MODS, heart failure, shock, vasoactive drugs and PCIS, cTnI and CK-MB concentrations have limited prognostic significance, and they were misjudged as confounding factors. However, they are useful for the assessment of prognosis at an early stage. The simultaneous elevations of CK-MB and cTnI concentrations within 24 h of admission to the PICU is an independent risk factor of shock, heart failure and MODS. Meanwhile, shock, heart failure and MODS are independently associated with mortality The possible reason is that the increase in the concentrations of CK-MB and cTnI indicates myocardial injury. Cardiac insufficiency and hemodynamic changes are likely to develop from the original disease, further aggravating the original disease and myocardial damage. This leads to the development of irreversible heart failure and MODS, which affects the prognosis.

### 4.7 Summary

Critically ill children without primary cardiac disease are susceptible to myocardial injury, with an incidence of 55.1%. Infectious diarrhea combined with moderate and severe dehydration is likely to cause myocardial injury in the PICU. Both CK-MB and cTnI are suitable for the early diagnosis and monitoring of myocardial injury in critically ill children without primary cardiac disease, and the combined detection of their elevated concentrations is more meaningful. Children with elevated cTnl and CK-MB concentrations are more likely to have shock, heart failure, and MODS. These conditions can lead to death. Higher cTnI and CK-MB concentrations are associated with a worse prognosis. Therefore, the combined detection of CK-MB and cTnI is crucial to improving the prognosis of critically ill children without primary cardiac disease and reducing the mortality rate. The outcomes from this study will facilitate early risk stratification for children with critical illness and ensure that those with elevated concentrations of biomarkers are monitored closely and treated aggressively. Further research is encouraged to validate the findings of this study.
