# Corrigendum: Significance of detecting cardiac troponin I and creatine kinase MB in critically Ill children without primary cardiac illness

**DOI:** 10.3389/fped.2024.1495374

**Published:** 2024-10-14

**Authors:** Yangyang Zhang, Yinyin Cao, Yi Xin, Yongming Liu

**Affiliations:** ^1^ Department of Pediatrics, Qingdao University Medical College Affiliated Yantai Yuhuangding Hospital, Yantai, Shandong, China; ^2^ Clinical Laboratory, Qingdao University Medical College Affiliated Yantai Yuhuangding Hospital, Yantai, Shandong, China

**Keywords:** primary cardiac disease, critical illness, myocardial injury, cardiac troponin I, creatine kinase, children

A Corrigendum on Significance of detecting cardiac troponin I and creatine kinase MB in critically Ill children without primary cardiac illness By Yangyang Zhang, Yinyin Cao, Yi Xin and Yongming Liu. (2024). Sec. Pediatric Critical Care. 12. doi: 10.3389/fped.2024.1445651

In the published article, there was an error in the article type. Instead of “ Review”, it should be “Original Research”.

The authors apologize for this error and state that this does not change the scientific conclusions of the article in any way. The original article has been updated.

