# Supplementary material for: Significance of detecting cardiac troponin I and creatine kinase MB in critically Ill children without primary cardiac illness
Source: Front Pediatr. 2024 Sep 2;12:1445651. doi: 10.3389/fped.2024.1445651 (PMC11402721; doi:10.3389/fped.2024.1445651)
Supplement: Supplementary file 1 [file Table1.pdf]

**Supplementary Table 1 Logistic regression analysis** Dependent variable: Shock								
Independent variable	B	S.E,	Wals	df	Sig.	Exp (B)	EXP(B)95% C.I.	
							Lower limit	Upper limit
Age	0.011	0.004	7.636	1	0.006	1.011	1.003	1.018
Gender	-0.463	0.340	1.850	1	0.174	0.630	0.323	1.226
Respiratory failure	0.805	0.328	6.034	1	0.014	2.237	1.177	4.252
PCIS	-0.091	0.020	19.99	1	<0.001	0.913	0.877	0.950
CK-MB/cTnI abnormal			17.39	2	<0.001			
Single elevated (1)	0.022	0.432	0.003	1	0.959	1.022	0.438	2.384
Double elevated (2)	1.396	0.381	13.43	1	<0.001	4.039	1.915	8.521
Constant	4.758	1.592	8.931	1	0.003	116.4		

**Supplementary Table 2 Logistic regression analysis** Dependent variable: Heart failure								
Independent variable	B	S.E,	Wals	df	Sig.	Exp (B)	EXP(B) 95% C.I.	
							Lower limit	Upper limit
Age	0.009	0.004	4.534	1	0.033	1.009	1.001	1.017

Gender	0.410	0.373	1.207	1	0.272	1.506	0.725	3.127
Respiratory failure	1.060	0.394	7.235	1	0.007	2.886	1.333	6.249
PCIS	-0.101	0.023	19.22	1	<0.001	0.904	0.864	0.946
CK-MB/cTnI abnormal			10.32	2	0.006			
Single elevated (1)	-0.033	0.508	0.004	1	0.948	0.967	0.357	2.618
Double elevated (2)	1.155	0.429	7.240	1	0.007	3.174	1.368	7.363
Constant	3.684	1.720	4.588	1	0.032	39.816		

Supplementary Table 3 Logistic regression analysis Dependent variable: MODS

Independent variable	B	S.E,	Wals	df	Sig.	Exp (B)	EXP(B) 的 95% C.I.	
							Lower limit	Upper limit
age	0.005	0.003	1.911	1	0.167	1.005	0.998	1.011
gender	0.134	0.283	0.223	1	0.637	1.143	0.656	1.991
PCIS	-0.060	0.018	11.57	1	0.001	0.942	0.910	0.975
CK-MB/cTnI abnormal			18.71	2	<0.001			

Single elevated (1)	0.161	0.313	0.265	1	0.607	1.175	0.636	2.168
Double elevated (2)	1.647	0.389	17.91	1	<0.001	5.190	2.421	11.13
Respiratory failure	1.472	0.294	25.04	1	<0.001	4.357	2.448	7.754
Constant	3.092	1.437	4.630	1	0.031	22.030		

Supplementary Table 4 Logistic regression analysis Dependent variable: Prognosis

Independent variable	B	S.E,	Wals	df	Sig.	Exp (B)	EXP(B) 95% C.I.	
							Lower limit	Upper limit
Age	0.008	0.003	6.122	1	0.130	1.008	1.002	1.015
Gender	0.500	0.280	3.187	1	0.074	1.649	0.952	2.856
PCIS	-0.082	0.018	21.46	1	<0.001	0.922	0.890	0.954
CK-MB/cTnI abnormal			4.435	2	0.109			
Single elevated (1)	0.460	0.327	1.975	1	0.160	1.584	0.834	3.010
Double elevated (2)	0.684	0.338	4.105	1	0.043	1.982	1.023	3.843
Respiratory failure	0.807	0.272	8.796	1	0.003	2.241	1.315	3.819
Vasoactive drugs	0.899	0.294	9.385	1	0.002	2.458	1.383	4.370
Constant	3.582	1.384	6.697	1	0.010	35.93		
